# Comparing nurse-led and rheumatologist-led care for rheumatoid arthritis patients: a systematic review and meta-analysis of randomized controlled trials

**DOI:** 10.1590/1980-220X-REEUSP-2024-0186en

**Published:** 2025-06-20

**Authors:** Weiwei Gu, Xiangfu Ding, Xu Han, Junjie Jiang, Hong Li

**Affiliations:** 1 Jilin University, China-Japan Union Hospital, Department of Gastrointestinal Surgery, Changchun, Jilin, China.; 2 Jilin University, The Second Norman Bethune Hospital, Department of Thyroid Surgery, Changchun, Jilin, China.; 3 Jilin University, The Second Norman Bethune Hospital, Department of Rehabilitation, Changchun, Jilin, China.; 4 Jilin University, China-Japan Union Hospital,Traditional Chinese Medicine Department, Changchun, Jilin, China.

**Keywords:** Arthritis, Rheumatoid, Primary Care Nursing, Rheumatologist, Effectiveness, Meta-Analysis, Artrite Reumatoide, Enfermagem de Atenção Primária, Reumatologistas, Efetividade, Metanálise

## Abstract

**Objective:**

This study aimed to compare nurse-led and rheumatologist-led management approaches for patients with rheumatoid arthritis, as previous studies yielded inconsistent results.

**Method:**

This study explored the PubMed, Embase, and Web of Science databases for research comparing these two therapy methods for individuals with rheumatoid arthritis. Outcomes analyzed were the Health Assessment Questionnaire, fatigue, stiffness, Disease Activity Score for 28 joints using C-reactive protein, Disease Activity Score for 28 joints, erythrocyte sedimentation rate, C-reactive protein, and visual analog scale pain score.

**Results:**

Six randomized controlled trials with 1,093 patients were included following the Cochrane Collaboration principles. The baseline parameters were similar between groups, except for the Disease Activity Score for 28 joints, satisfaction levels, and disease duration. Pooled analyses demonstrated that the nurse-led care group tended to improve relevant assessment indicators relative to the rheumatologist-led care group.

**Conclusion:**

The meta-analysis results suggest that rheumatoid arthritis patients in nurse-led care tend to have a better functional status and disease activity trend than those in rheumatologist-led care.

## RESUMO

**Objetivo:**

Este estudo tem como objetivo comparar a eficácia de dois modelos de cuidado no manejo de pacientes com artrite reumatoide: um liderado por enfermeiros e outro por reumatologistas, considerando que estudos anteriores apresentam resultados contraditórios sobre a eficácia dessas abordagens.

**Métodos:**

Foi realizada uma busca nas bases de dados PubMed, Embase e Web of Science para identificar estudos que compararam a eficácia desses dois modelos de cuidado em pacientes com artrite reumatoide. Os desfechos analisados incluíram o Questionário de Avaliação de Saúde (Health Assessment Questionnaire), nível de fadiga, rigidez matinal, Escore de Atividade da Doença em 28 Articulações baseado na Proteína C Reativa (DAS28-CRP), Escore de Atividade da Doença em 28 Articulações (DAS28), velocidade de hemossedimentação, proteína C reativa e Escala Visual Analógica para dor.

**Resultados:**

Com base nos princípios da Colaboração Cochrane, foram incluídos seis ensaios clínicos randomizados, abrangendo um total de 1.093 pacientes. Exceto pelos escores de atividade da doença em 28 articulações, nível de satisfação e duração da doença, as características basais dos pacientes foram comparáveis entre os grupos. A análise combinada indicou que o modelo de cuidado liderado por enfermeiros apresentou uma tendência de melhora nos desfechos clínicos avaliados, em comparação com o modelo liderado por reumatologistas.

**Conclusão:**

Os achados desta meta-análise sugerem que pacientes com artrite reumatoide sob cuidados liderados por enfermeiros apresentam tendência a melhores resultados em termos de funcionalidade e atividade da doença, em comparação com aqueles sob cuidados liderados por reumatologistas.

## INTRODUCTION

Rheumatoid arthritis (RA) is a chronic inflammatory disease characterized by symptoms that include joint inflammation, synovial hyperplasia, and the irreversible loss of bone tissue^(1,2)^. In 2020, an estimated 17.6 million individuals globally experienced rheumatoid arthritis^(3,4)^. Rheumatoid arthritis can impact people of any age, including men, women, and children; however, it is two to three times more prevalent in women. Its incidence increases with age, typically between sixty and seventy years old^(5)^. Most RA patients require ongoing medical and physiotherapeutic care to arrest or slow the progression of this disease, contributing to high treatment-related costs^(6)^. However, the quality of life for millions of people worldwide is negatively impacted by these costs, as well as the high rates of RA-related morbidity and mortality^(6)^. In addition to the substantial indirect costs that RA patients suffer due to lost productivity, novel therapies are also very costly^(6,7,8)^. When not effectively treated, RA patients can experience decreased functional performance, high rates of morbidity, and a dramatically shortened life expectancy^(9,10)^. With the rising prevalence of chronic diseases and the extended lifespans of affected individuals, there is an increasing demand for community and hospital-based care, necessitating that nurses frequently assume medical responsibilities traditionally held by physicians or specialists^(1,2)^. Nurse-led care (NLC) is considered a valuable supplement to physician-led care that can improve the well-being and quality of life of patients suffering from cancers and chronic diseases^(4)^. NLC entails a holistic person-centred care strategy promoted by the World Health Organization as a key facet of high-quality healthcare efforts^(11,12)^, contributing to greater satisfaction among nurses and patients concerning the quality of care^(11)^.

The in-hospital and clinical treatment of RA patients is the responsibility of rheumatologists. However, the number of these specialists is anticipated to decline in the future^(1,2)^. As a result, rheumatology nurses have become increasingly involved in completing tasks performed by rheumatologists, such as prescribing drugs and administering joint injections^(1)^. These nurses also play key roles as members of multidisciplinary teams^(1)^.

The findings of the 6 RCTs are inconsistent with previous research, suggesting further analysis. This study assessed the prognostic implications of nurse-led versus rheumatologist-led care for patients with rheumatoid arthritis. A randomized trial published in 2015 demonstrated that improvements in rheumatoid arthritis patient health assessment questionnaire (HAQ) scores and overall costs were similar, regardless of whether patients received nurse-led care (NLC) or rheumatologist-led care (RLC)^(1)^. Other groups have yielded inconsistent results^(1)^. In total, 6 randomized controlled trials (RCTs) focused on this topic were published from 2020-2023^(13,14,15,16,17,18)^.

This article aims to conduct a data analysis and systematic review of the existing clinical randomized controlled trials (RCTs) to obtain the latest evidence-based medical evidence to confirm the advantages and disadvantages of the two treatment modalities. This study aims to conduct a thorough analysis and systematic assessment of the existing clinical RCT data on RA. This study will consolidate disparate research findings and derive the most recent and reliable evidence-based medical data through meticulous statistical analysis and a comprehensive literature evaluation. This study will specifically compare the efficacy of the two therapy modalities in enhancing the conditions of RA patients. This assessment will encompass various dimensions, including short-term symptom relief (e.g., reduction in joint pain and swelling), long-term disease control (such as maintenance of joint function and delay in radiographic progression), frequency and severity of adverse reactions, and improvement in the patient’s quality of life, to elucidate their respective strengths and weaknesses. Finally, the outcomes of this study should offer a strong evidence-based foundation for the effective clinical practice of RA patient care. Developing customized treatment plans will help medical staff make more scientific and exact decisions, optimizing RA patients’ total treatment outcomes and prognoses. This will help the field of rheumatoid arthritis treatment improve and advance and provide useful reference guidelines and additional recommendations for conducting subsequent relevant studies.

Previous studies on the efficacy of NLC relative to that of RLC for RA patients have yielded inconsistent outcomes. This study was thus designed to compare RLC and NLC as approaches to RA patient management.

## METHOD

### Study Design

**Figure 1:**
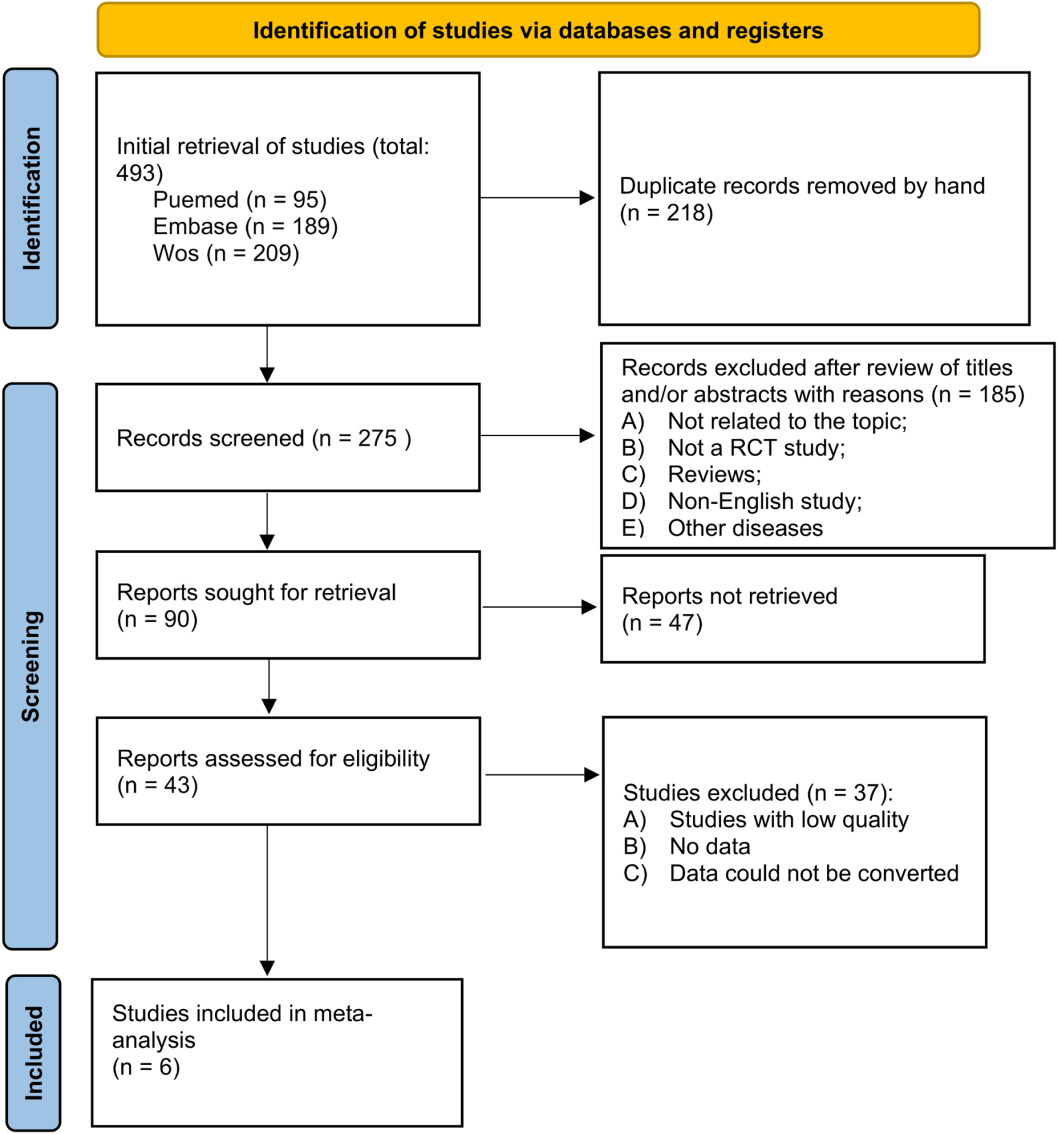
Flow diagram of literature inclusion and exclusion process.

Using the Cochrane Collaboration (Preferred Reporting Items for Systematic Reviews and Meta-analysis) 2020 statement as a guide, studies examining the costs and effectiveness of RLC and NLC in the treatment of RA patients were found and used to perform a systematic review and meta-analysis of RCTs^(19)^. This analysis was prospectively registered in the PROSPERO database (CRD42023403128), and the PRISMA 2020 checklist is provided in Figure 1.

### Study Eligibility

Eligibility for inclusion in this meta-analysis required studies to meet the following criteria: (1) be RCTs; (2) focus on patients with RA or chronic inflammatory arthritis; (3) compare non-linear control (NLC) and RLC; (4) report at least one of the following outcomes: Disease Activity Score for 28 joints (DAS28), DAS28 based on C-reactive protein (DAS28-CRP), Health Assessment Questionnaire (HAQ), Visual Analog Scale (VAS) for pain, satisfaction, disease duration, or clinical outcomes; and (5) provide proper information for the calculation of weighted mean difference (WMD) or risk ratio (RR) values. This analysis excluded research that was not published in English, unpublished papers, case reports, letters, reviews, conference abstracts, editorial comments, and studies involving pediatric patients. Furthermore, epidemiological analyses and studies that only examined the costs or effectiveness of RLC or NLC separately were not included.

P: Patients with RA or chronic inflammatory arthritis

I: NLC

C: RLC

O: DAS 28, DAS 28-CRP, HAQ, VAS Pain, Satisfaction, Disease duration, or Clinical outcomes, etc

S: RCT

### Literature Search Strategy

We searched PubMed from 1964 to February 10, 2023, EMBASE from 1974 to February 10, 2023, and Web of Science from 1945 to February 10, 2023. The related keywords and medical subject heading were set as search terms: (Nurses’ Practice Patterns) OR (Nurse’s Practice Patterns)) OR (Nurse Practice Patterns)) OR (Nurse’s Practice Pattern)) OR (Practice Pattern, Nurse’s)) OR (Practice Patterns, Nurse’s)) OR (Nurse-Led Clinics)) OR (Clinic, Nurse-Led)) OR (Clinics, Nurse-Led)) OR (Nurse Led Clinics)) OR (Nurse-Led Clinic)) OR (nurse-led)) OR (“Practice Patterns, Nurses’”[Mesh])) AND (((Rheumatoid Arthritis)) OR (“Arthritis, Rheumatoid”[Mesh])) (Supplementary File 1). All relevant research references were carefully examined to identify additional studies of potential relevance. Furthermore, the reference list of each qualified study was examined personally. Two researchers independently conducted a search and evaluation of the included studies. All differences in the literature review were resolved through consensus.

### Data Extraction

Data extraction was conducted independently by two researchers, GWW and HX. All co-authors achieved a consensus to address any disputes. Data obtained from the chosen studies encompassed the following: first author, year of publication, study design, origins of enrolled participants, number of patients in each group, patient ages, gender ratio in the patient population, and clinical data, including mean disease duration, DAS 28, DAS 28-CRP, HAQ, and VAS. Discomfort, contentment, exhaustion, stiffness, inflamed and sensitive joints. For continuous variables reported as medians and ranges or interquartile ranges, mean ± standard deviation values were instead calculated with a previously validated mathematical approach^(20,21)^. Attempts were undertaken to reach the corresponding authors of studies lacking complete data. Data were independently extracted by two investigators, with any disputes resolved by a third investigator.

### Statistical Analysis

Review Manager 5.3 (Cochrane Collaboration, Oxford, UK) was used to conduct pooled analyses. Continuous and categorical variables were compared using WMD and OR values with corresponding 95% confidential intervals (CIs). The chi-squared (χ^2^) test (Cochran’s Q) and the inconsistency index (*I* ^2^) were used to evaluate heterogeneity among studies^(22)^, with a χ^2^ P < 0.05 or an *I* ^2^ > 50% being indicative of significant heterogeneity. The impact of individual studies on pooled results showing significant heterogeneity was also examined using one-way sensitivity analyses, and funnel plots developed during Review Manager 5.3 were visually inspected to identify potential publication bias. When heterogeneity was identified, pooled WMD or OR values were estimated using a random-effect model, while a fixed-effect model was used otherwise.

## RESULTS

### Study Selection

Figure 1 shows a flow chart that describes the study selection procedure. A total of 95, 189, and 209 potentially relevant publications were first obtained from Web of Sciences, EMBASE, and PubMed; 218 of these were removed as duplicates. After carefully examining the corresponding abstracts and titles, 30 of the 277 papers were selected for full-text review. Finally, 6 studies enrolling 1,093 patients (NLC: 543; RLC: 550) were selected for inclusion in this meta-analysis^(13–18)^, all randomized controlled trials. For a summary of the characteristics of these studies, see Table 1 and Figure 1.

**Table 1:** Baseline characteristics of include studies and methodological assessment – Changchun, China, 2025.

Authors (Publish year)	Study period	Country (City)	Study design	Patients (n)	Median follow-up (months)
				Nurse-led care/ Rheumatologist-led care	
Larsson et al. (2014)	2009/10–2011/08	Sweden (Lund)	RCT	47/50	12
Ndosi et al. (2014)	–	UK (Leeds; Staffordshire; Stoke-on-Trent)	RCT	91/90	–
Larsson et al. (2015)	2009/10–2010/08	Sweden (Lund)	RCT	47/50	12
Hoeper et al. (2021)	2018/01–2018/08	Germany (Hannover; Osnabruck; Planegg; Erlangen; Hildesheim; Mainz; Bad Kreuznach; Bad Pyrmont)	RCT	111/113	12
Kwok et al. (2022)	–	China (Hong Kong)	RCT	140/140	24
Wang et al. (2018)	2015/01–2015/11	China (Wenhai)	RCT	107/107	–

RCT, Randomized controlled trial

The Cochrane risk assessment technique was used to assess the quality of the included literature. Random number table allocation was used in all 6 of the included investigations, which was considered low risk. Blinding was used to assign individuals to 6 studies regarded as low-risk. The remaining investigations were classified as low-risk and detailed the precise allocation process.

**Figure 2:**
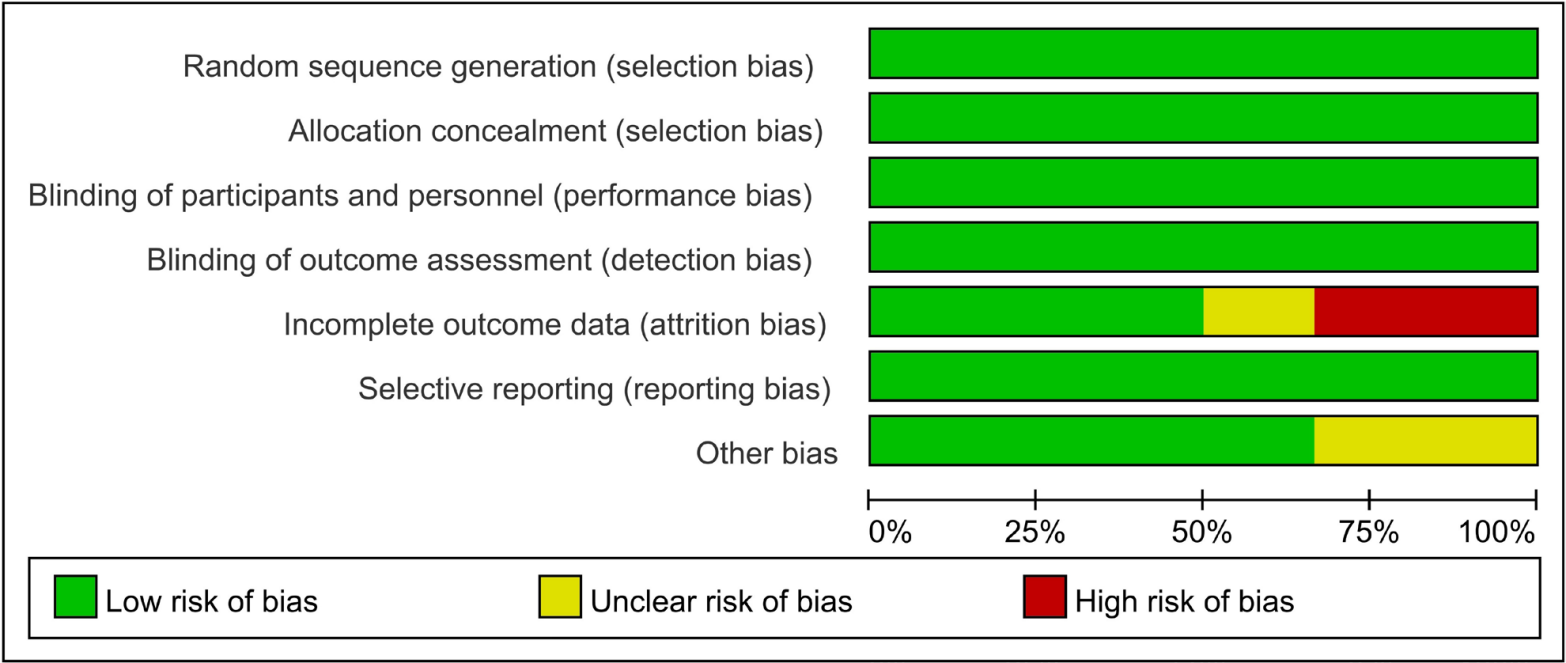
Risk of bias graph.

All 6 studies used the envelope method for allocation concealment, which was assessed as low risk. Double-blinding was mentioned in all six studies, and they were deemed low risk. Two studies^(13,14)^ had dropouts with corresponding explanations and were assessed as high-risk. One study^(15)^ had not completed outcome data and was assessed as low risk. The remaining studies possessed complete outcome data and were assessed as low-risk. All studies were registered, indicating that selective reporting was evaluated and determined to pose a low risk. Two studies^(13,14)^ were found to have other biases and were evaluated as low-risk (Figure 2).

### Baseline Demographic Characteristics

No significant differences were noted between these groups concerning baseline age (WMD: –0.18; 95% CI: –1.26, 0.91; p = 0.75), gender (male/total, RR: 1.12; 95% CI: 0.92, 1.37; p = 0.26), DAS28-CRP (WMD: –0.09; 95% CI: –0.20, 0.01; p = 0.08), HAQ (WMD: –0.09; 95% CI: –0.17, 0.00; p = 0.05), VAS pain scores (WMD: –0.16; 95% CI: –0.90, 0.59; p = 0.68), fatigue (WMD: 0.26; 95% CI: –0.61, 1.14; p = 0.56), stiffness (WMD: –1.43; 95% CI: –7.17, 4.30; p = 0.62), swollen joints (WMD: –0.06; 95% CI: –0.21, 0.09; p = 0.44), or tender joints (WMD: –0.09; 95% CI: –0.36, 0.18; p = 0.50). There were, however, significant differences concerning baseline DAS28 (WMD: –0.16; 95% CI: –0.32, 0.01; p = 0.03), satisfaction (WMD: 0.28; 95% CI: 0.10, 0.46; p = 0.002) and disease duration (WMD: 0.60; 95% CI: 0.02, 1.19; p = 0.04) values (Table 2).

**Table 2:** Demographics and clinical characteristics of included studies – Changchun, China, 2025.

Outcomes	Studies	No. of patients	WMD or RR	95% CI	p-value	Heterogeneity			
		Nurse-led care/ Rheumatologist-led care				Chi^2^	df	p-value	*I* ^2^ (%)
Age (years)	6	543/550	–0.18	[–1.26, 0.91]	0.75	3.67	5	0.62	0
Gender (male)	6	543/550	1.12	[0.92, 1.37]	0.26	1.24	5	0.94	0
DAS 28	4	286/293	–0.16	[–0.32, –0.01]	0.03*	0.25	3	0.97	0
DAS 28-CRP	3	298/303	–0.09	[–0.20, 0.01]	0.08	0.09	2	0.96	0
HAQ	4	325/330	–0.09	[–0.17, 0.00]	0.05	3.20	3	0.36	6
VAS Pain (0–100mm)	5	496/500	–0.16	[–0.90, 0.59]	0.68	6.81	4	0.15	41
Satisfaction (0–10)	2	158/163	0.28	[0.10, 0.46]	0.002*	0.16	1	0.68	0
Disease duration (years)	6	543/550	0.60	[0.02, 1.19]	0.04*	3.65	5	0.60	0
Fatigue (VAS score)	3	309/310	0.26	[–0.61, 1.14]	0.56	1.83	2	0.40	0
Stiffness	2	198/197	–1.43	[–7.17, 4.30]	0.62	0.40	1	0.52	0
Swollen joints	2	218/220	–0.06	[–0.21, 0.09]	0.44	0.01	1	0.91	0
Tender joints	2	218/220	–0.09	[–0.36, 0.18]	0.50	0.78	1	0.38	0

^*^ Statistically significant.

DAS28, disease activity score in 28 joints; WMD, weighted mean difference; RR, risk ratio; CI, confidence interval; HAQ, Health Assessment Questionnaire; VAS, visual analogue scale.

### Haq Scores

**Figure 3:**
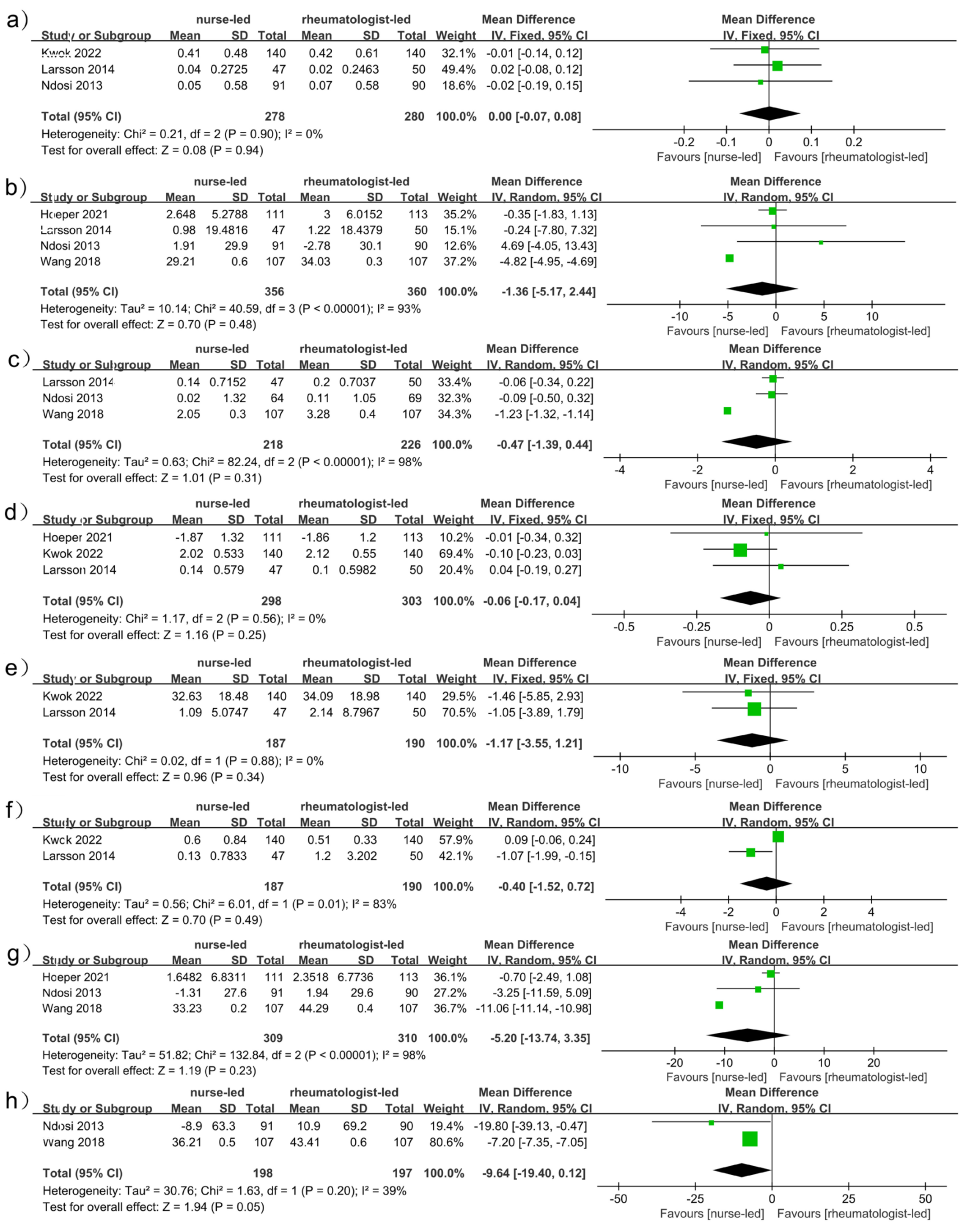
Forest plot of the study evaluating the difference in the improvement of HAQ (A), VAS Pain (B), DAS28 (C), DAS28-CRP (D), ESR (E), CRP (F), Fatigue (G), and Stiffness (H) between RLC and NLC for patients with RA.

HAQ scores were reported in 3 studies enrolling 558 patients (NLC: 278, RLC: 280)^(13,15,17)^. There was no significant heterogeneity (*I* ^2^ = 0%, p = 0.90), and no significant difference was found when comparing these two patient groups following treatment (WMD: 0.00, 95% (CI: –0.07 to 0.08; *p* = 0.94) (Figure 3A).

### Vas Pain Scores

**Figure 4:**
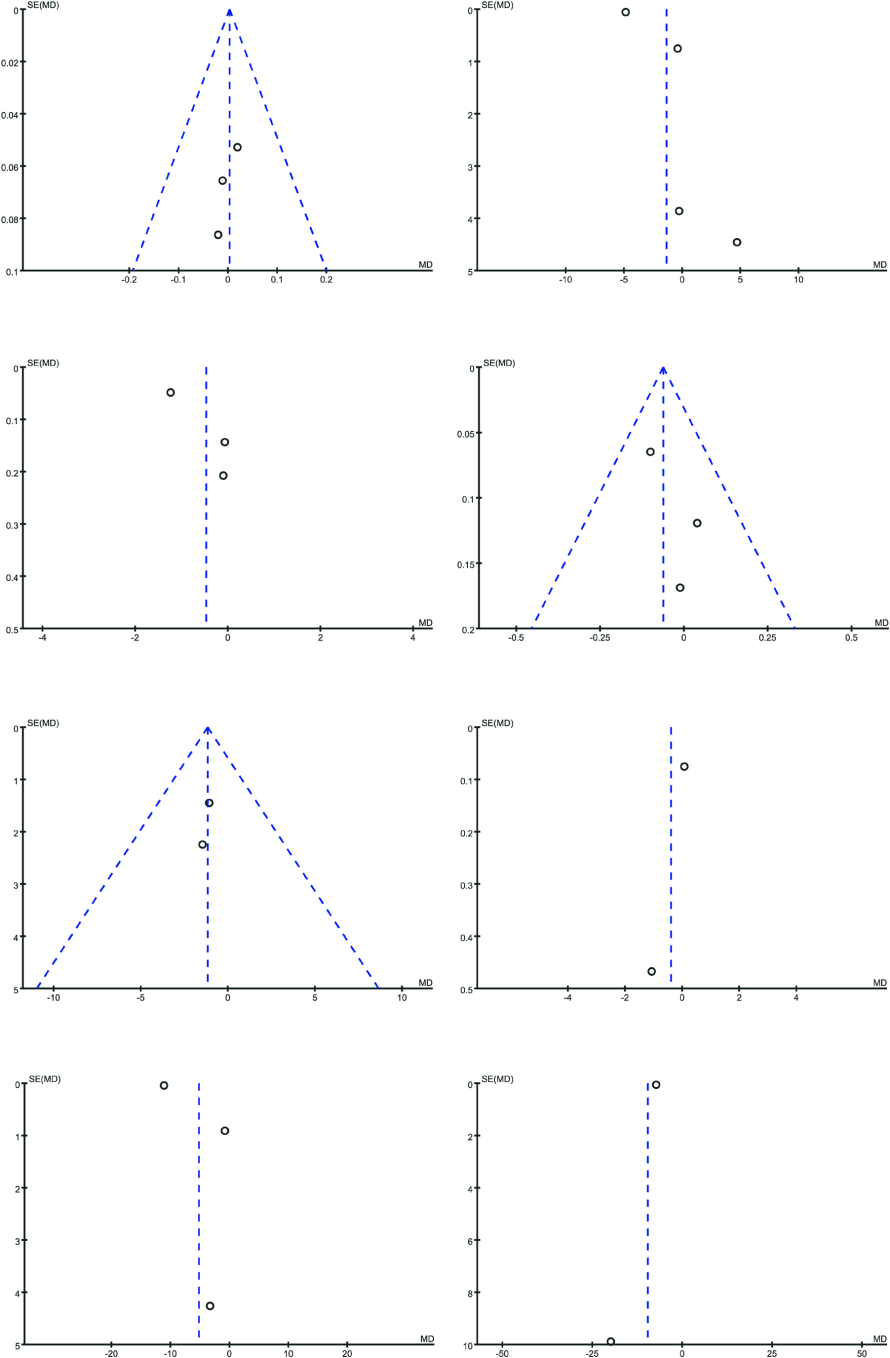
Funnel plots for HAQ (A), VAS Pain (B), DAS28 (C), DAS28-CRP (D), ESR (E), CRP (F), Fatigue (G), and Stiffness (H) between RLC and NLC for patients with RA.

Changes in VAS pain scores between groups were reported in 4 studies enrolling 716 patients (NLC: 356, RLC: 360)^(13,15,16,18)^. No significant differences in VAS scores were noted when comparing the NLC and RLC groups (RR: –1.36; 95% CI: –5.17, 2.44; *p* = 0.48), although there was evidence of significant heterogeneity (*I* ^2^ = 93%, *p* < 0.00001) (Figure 3B) and publication bias (Figure 4B).

### DAS28 Scores

DAS28-CRP data were reported in 3 RCTs enrolling 601 patients (NLC: 298, RLC: 303)^(15–17)^. Pooled analyses revealed similar improvements in DAS28-CRP scores in the NLC and RLC groups (WMD: –0.06; 95% CI: –0.17, 0.04; *p* = 0.25) (Figure 3D), with no evidence of heterogeneity (*I* ^2^ = 0%, *p* = 0.56) or publication bias (Figure 4D).

### DAS28-CRP Scores

DAS28-CRP data were reported in 3 RCTs enrolling 601 patients (NLC: 298, RLC: 303)^(15–17)^. With no indication of heterogeneity (*I* ^2^ = 0%, *p* = 0.56) or publication bias, pooled analysis showed comparable increases in DAS28-CRP scores in the NLC and RLC groups (WMD: –0.06; 95% CI: –0.17, 0.04; *p* = 0.25; Figure 3D) (Figure 4D).

### ESR

Erythrocyte sedimentation rate (ESR) related data were reported in 2 studies enrolling 377 patients (NLC: 187, RLC: 190)^(15,17)^. Pooled analyses revealed no significant differences in ESR values in the NLC group (WMD: –1.17; 95% CI: –3.55, 1.21; *p* = 0.34), and there was no evidence of significant heterogeneity (^2^ = 0%, *p* = 0.88) (Figure 3E). Slight publication bias was detected through visual inspection of funnel plots (Figure 4E).

### CRP

C-reactive protein (CRP) levels were analyzed in 2 studies enrolling 377 patients (NLC: 187; RLC: 190)^(15,17)^. Pooled analyses revealed no significant increase in CRP levels in the NLC group (RR: -0.40; 95% CI: –1.52, 0.72; *p* = 0.49). Significant heterogeneity (*I* ^2^ = 83%, *p* = 0.01) (Figure 3F) and slight publication bias were detected (Figure 4F).

### Fatigue

Fatigue was an analyzed endpoint in 3 studies enrolling 619 patients (NLC: 309; RLC: 310)^(13,16,18)^. Significant heterogeneity (*I* ^2^ = 98%, *p* < 0.00001) (Figure 3G) was detected in pooled analyses, which revealed a trend towards improved fatigue in the NLC group (RR: –5.20; 95% CI: –13.74, 3.35; *p* = 0.23). Funnel plots revealed publication bias and detected heterogeneity (Figure 4G).

### Stiffness

Stiffness was analyzed in 2 studies enrolling 395 patients (NLC: 198; RLC: 197)^(13,18)^. Pooled analyses revealed no significant improvements in stiffness in the NLC group relative to the RLC group (RR: –9.64; 95% CI: –19.40, 0.12; *p* = 0.05), with slight heterogeneity having been detected (*I* ^2^ = 39%, *p* = 0.20) (Figure 3H).

### Sensitivity Analyses

**Figure 5:**
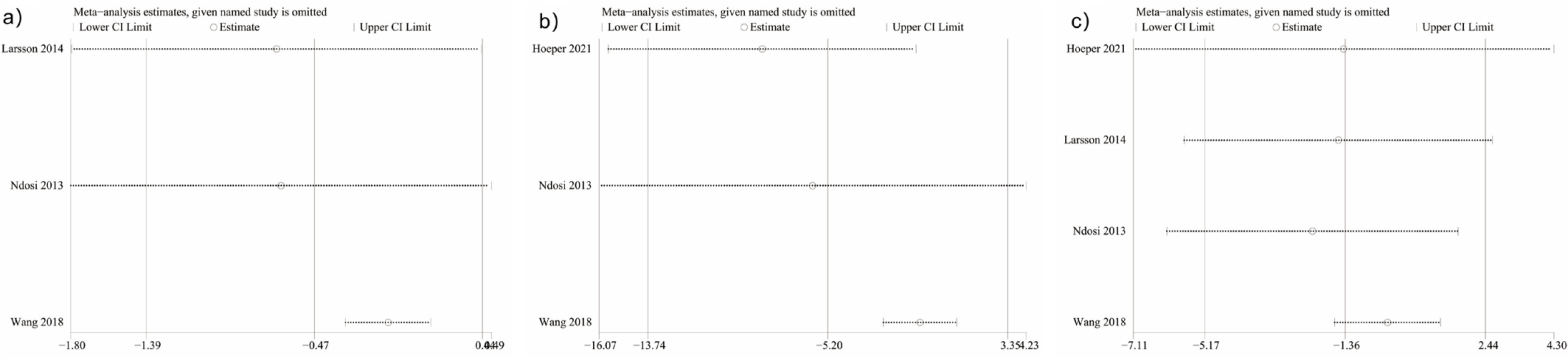
Sensitivity analysis of DAS28 (A), Fatigue (B), and VAS Pain (C) between RLC and NLC for patients with RA.

One-way sensitivity analyses were employed to analyze pooled DAS28, VAS pain score, and tiredness outcome comparisons by systematically excluding individual studies from the pooled analysis and evaluating the effect on the pooled relative risk (RR). The RR values remained constant after excluding any specific study for all three outcomes (Figure 5A–C). When the study conducted by Wang et al. in 2018^(18)^ was excluded, however, significant heterogeneity was no longer detected for pooled analyses of fatigue (*I* ^2^ = 0%, p = 0.56), DAS28 (*I* ^2^ = 0%, p = 0.91), or VAS pain scores (*I* ^2^ = 0%, p = 0.54), indicating that this study was the primary source of heterogeneity.

## DISCUSSION

The present meta-analysis was developed to compare the efficacy of RLC and NLC for RA patients based on differences in outcomes, including HAQ, DAS28, DAS28-CRP, ESR, CRP, fatigue, stiffness, and VAS pain score values between these groups^(23,24,25,26,27)^. NLC-based management for RA patients has previously been linked to improved opinions of their care and clinical outcomes^(28,29,30,31)^. There is insufficient data to reach definitive conclusions about the feasibility, efficacy, or relevance of NLC for this patient population^(28)^. RLC and NLC appear to be comparable, cost-effective approaches to RA patient management. Therefore, the current study was the most recent and largest pooled analysis focused on this topic, synthesizing data from 6 RCTs enrolling 1,093 RCT patients and producing several important insights. These analyses revealed that patients who underwent RLC showed a trend toward better clinical outcomes than patients who underwent NLC. However, there were no significant differences in outcomes between these two groups. RA is a chronic inflammatory disease that contributes to higher rates of mortality among affected patients^(32)^, in addition to causing significant adverse effects on functional status and quality of life owing to symptoms including generalized swelling, tenderness, and a higher risk of fractures^(33,34)^. The life expectancy of RA patients is reduced by an estimated 3–10 years relative to the general population, with a ~50% higher risk of premature death^(35)^. Although the exact etiological foundation of RA is still unknown, genetic and environmental factors contribute to its incidence^(36,37)^. Many different factors have been linked to the onset and progression of RA, including age, gender, genetic factors, hormone levels, ethnicity, socioeconomic factors, infections, smoking status, urbanization, and pollutant exposure^(32,38)^. The HAQ provides an effective means of assessing the quality of life and functional status of RA patients^(37,39)^, and this gold-standard analytical tool has thus been translated into many different languages^(40,41)^. The 20-item HAQ was designed as a broad instrument for assessing patient populations rather than solely addressing RA patients. It has items about 8 domains used for evaluating activities of daily living, including walking, reaching, dressing, gripping, and eating^(41,42)^. This scale has been established as a reliable and valid tool for RA patient assessment^(43)^. As a result, it was used to evaluate the functional status of RA patients, demonstrating comparable improvements in functional status for those who had RLC or NLC.

The DAS provides a quantitative tool that can be used to monitor disease activity and therapeutic responses in patients with RA^(44)^. This scale incorporates data about tender joints, swollen joints, ESR values, and CRP levels, together with an overall view of the reported general health of the patient^(45)^. The DAS is often used in the clinic when assessing RA severity, although it has not been established as the gold standard tool for use in this context^(46)^. One variable cannot fully encapsulate the disease activity in a given RA patient^(47)^. However, in clinical studies and standard clinical practice, the DAS/DAS28 has been used to assess the disease activity of RA patients both individually and in groups. DAS28 and DAS28-CRP parameters were used to evaluate the effectiveness of RLC- and NLC-based interventional techniques in the current study, and the results showed that both improved comparably in both RA patient groups. No significant differences in the DAS28 scores of RA patients undergoing NLC have previously been reported relative to those associated with RLC at 12 months^(15,48,49)^. However, NLC has been reported to show significant superiority after 2 years^(48)^.

In some studies, DAS28 scores have been reported to significantly improve in inflammatory arthritis patients at 9 months or RA patients at 12 months managed by NLC compared to RLC^(18,50)^. In comparison, other studies have failed to detect any such significant differences at 13-month^(18)^, 21-month^(50)^, or 24-month follow-up time points^(51)^. Similar ESR, CRP, and VAS pain score values were noted in both groups. When computing DAS or DAS28 scores, CRP levels can be used instead of ESR values^(52)^. CRP provides greater accuracy as an indicator of inflammatory activity and rises more rapidly following exposure to inflammatory stimuli^(53)^. Chew et al.^(54)^ found that disease activity in most RA patients remained stable among patients who received NLC with slight increases in mean DAS28 scores. Comparable improvements in stiffness and fatigue were observed in both groups, in contrast to the findings of a previous meta-analysis, which indicated that NLC could not alleviate fatigue in RA patients^(31)^. However, no significant differences in outcomes were noted when comparing the NLC and RLC groups^(55)^. The basis for these results remains uncertain. However, recognition of the significance of NLC is increasing among the medical community, and it presents potential benefits in mitigating weariness among RA patients through nurse-led educational and psychosocial support treatments. NLC yields benefits that include greater continuity of care and the ability of patients to access care while remaining close to home^(56)^. In individuals with chronic diseases, NLC-based care models have been established in the USA, Canada, and Australia^(13)^. NLC strategies enable rheumatologists to work more effectively while experiencing lower stress levels^(15)^. One multi-center RCT reported that NLC was superior to RLC for RA patient satisfaction and consultation costs^(13)^. Furthermore, compared to patients who received RLC, the current data indicate that patients who received NLC showed larger gains in their functional status and clinical outcomes. In the case of Wang et al.’s study, several factors may contribute to the observed variability. Many factors can influence heterogeneity in the results, particularly in measures like VAS pain, fatigue, and DAS28.

The research conducted by Wang et al. established distinct inclusion and exclusion criteria. Patients who were recently diagnosed, displayed severe disability, or presented with unstable disease were excluded. This selection process may have resulted in a specific patient population with unique characteristics, which could differ from those in other studies, potentially influencing outcomes and contributing to heterogeneity.

Applying tools such as the DAS28 and VAS to assess pain and fatigue can show variability in interpretation and usage. Despite training, assessors can vary their methods for counting affected joints in DAS28 or evaluating pain and fatigue on the VAS. This may result in variations in recorded scores and contribute to heterogeneity relative to other studies. Standardizing terminologies in the management of rheumatoid arthritis can enhance communication, improve research comparability, and lead to better patient outcomes for healthcare practitioners. The standardization is essential due to the evolving roles of nurses in rheumatology care, necessitating clear definitions and standards for NLC and RLC models^(16,18,57,58)^.

The study was carried out within a healthcare system in China, which could demonstrate distinct characteristics, including variations in medication availability and accessibility, differences in healthcare service organization, and cultural factors affecting patient-provider interactions. The contextual factors may interact with the NLC and RLC models in ways that differ from other regions or healthcare systems, resulting in heterogeneity in the findings relative to studies conducted in different contexts.

In summary, the variability in the findings of Wang et al.’s study, particularly regarding measures such as VAS pain, fatigue, and DAS28, may result from a complex interplay of various factors associated with study design, intervention implementation, data collection, and the broader healthcare context. Understanding these potential sources of heterogeneity enhances the interpretation of results and facilitates comparisons with other field studies.

Despite data aggregation from multiple studies, the absence of statistical significance in outcomes such as fatigue and stiffness in the meta-analysis may be due to various clinical and statistical factors. Fatigue and stiffness in RA are multifactorial symptoms. They can be influenced not only by the disease activity but also by various other factors, such as comorbidities, lifestyle, and psychological factors. Different studies might have had varying proportions of patients with these confounding factors, which could have masked the true effect of the NLC or RLC models on fatigue and stiffness.

Substantial heterogeneity may have been introduced by the techniques employed to quantify stiffness and fatigue in various trials. Measures of stiffness duration and the VAS for tiredness are subjective and vulnerable to biases in reporting and patient perception. It is possible that slightly different versions of these assessment instruments or patient instructions for how to rate symptoms were used in other studies. This measurement inaccuracy may have decreased the possibility of finding a statistically significant difference and increased data variability. Wang et al.^(59)^ conducted randomized trials in Chinese patients with RA to evaluate the clinical effectiveness and cost-effectiveness of NLC compared to RLC. Their preliminary findings indicated that RA patients managed by NLC may have better clinical outcomes and more cost-effective care. A study^(60)^ implemented a patient-initiated review system for people with RA, which improved patient satisfaction and resource use. In a study^(61)^, the outcome and cost-effectiveness of community-based NLC were compared with rheumatologist-led outpatient care. The study reported results from regression analyses and suggested that NLC in the community could be a cost-effective option for managing RA patients.

Furthermore, Lopatina et al.^(62)^ compared healthcare resource utilization and costs in stable patients with RA under nurse-led and rheumatologist-led care delivery models. Literature indicates that NLC may provide superior clinical outcomes and cost-effective care for patients with RA compared to rheumatologist-led care. Adopting NLC strategies could enhance patient satisfaction, resource utilization, and the overall quality of care for RA patients. Further research and implementation strategies are advised, especially in Africa and the Middle East^(29)^.

This study is subject to several strengths and limitations. Importantly, this analysis was based on 6 RCTs in which patients were randomly assigned to care groups, and complete care data were available^(13-18)^, strengthening the credibility of the resultant conclusions. However, the follow-up intervals for these studies differed substantially, potentially resulting in some degree of bias when interpreting the pooled results. This study used databases like PubMed, Embase, and Web of Science to search only for published articles. This study cannot ensure the collection of unpublished, grey, and currently submitted articles for comprehensive analysis. Therefore, data inaccuracies may necessitate further examination and rectification in future research. Future research should involve additional high-quality experiments that consider follow-up intervals, dependent upon the publication of more studies on this topic. This meta-analysis did not include unpublished studies, which may have led to the omission of relevant data.

Awareness of cultural nuances enables nurses to tailor interventions effectively, enhancing patient engagement and adherence to treatment, thus improving health outcomes^(63)^. Nurse-led care models can be more readily integrated into RA treatment in countries with strong healthcare systems, facilitating comprehensive management strategies encompassing support, monitoring, and education. By examining these variations, researchers can identify best practices that may be adapted for application in diverse healthcare settings, ensuring that nurse-led care is customized to meet the specific needs of the population^(64)^. Younger patients might favor more technologically advanced methods, like telehealth consultations with nurses. However, older patients might gain more from face-to-face meetings with rheumatologists. Knowing these preferences can help create individualized care plans that improve patient outcomes and satisfaction^(65,66)^. By looking at these factors, researchers can better understand how socioeconomic status interacts with care models to influence patient outcomes^(67)^. Understanding regional variations facilitates resource allocation and ensures that healthcare systems are equipped to meet the specific needs of their populations^(68)^.

The resource availability across various healthcare systems differs significantly. In a resource-rich healthcare system, the NLC model has the potential to effectively integrate multidisciplinary teams, including physical therapists and dietitians, thus enhancing comprehensive patient care and positively influencing patient prognosis. In a resource-limited healthcare system, the RLC model may strengthen diagnosis and treatment decisions owing to the professional authority of physicians; however, it may fall short in delivering adequate non-drug interventions, including patient education and psychological support.

Interestingly, a meta-analysis found no discernible difference in physician-led and nurse-led follow-up results, indicating that nurse-led care can successfully manage RA patients with low disease activity as traditional rheumatologist-led care^(69,70)^. A pragmatic, non-randomized study evaluated the cost-effectiveness of community-based NLC versus RLC. Findings indicated that NLC was associated with lower annual costs while maintaining clinical outcomes. In particular, the total annual cost of rheumatology care for the NLC group was significantly less than that of the RLC group, suggesting that NLC provides effective management with reduced medical expenses^(61,71)^. It has also been observed that the use of disease-modifying antirheumatic medications (DMARDs) is growing; between 1998 and 2009, their use increased significantly. This pattern indicates a larger movement toward aggressive treatment approaches seeking low disease activity or remission, essential for long-term patient outcomes^(72,73)^. There are some limitations when it comes to excluding nursing databases for reference. In-depth research findings with distinct viewpoints or particular nursing concerns may be missed, and other extensive databases may not frequently incorporate these findings. Nursing databases offer succinct presentations of specific cases, nursing experiences, and other information pertinent to nursing practice. The absence of these elements may lead to an incomplete understanding of certain nursing phenomena and hinder the capacity to effectively address the diversity and specificity within the nursing field, compromising the comprehensiveness and applicability of meta-analyses concerning nursing research topics. Publication bias may be indicated by a funnel plot showing clear asymmetry, evidenced by the absence of specific study points on one side. Various strategies exist to mitigate the impact of publication bias. This involves carefully identifying unpublished studies to address any deficiencies, such as by exploring the clinical trial registry or contacting the study authors to obtain unpublished data. Upon the exclusion of potentially biased research, conduct a sensitivity analysis to evaluate the robustness of the results. The efficacy and constraints of the six randomized controlled trials assessing the effects of NLC and RLC on the prognosis of RA patients can be more comprehensively analyzed through the aforementioned detailed examination of research diversity and publication bias, providing valuable references for clinical practice and future investigations. Not publishing the protocol of RSL leads to lack of transparency and reduced reproducibility. It increases bias risks like selective reporting. In clinical practice, this generates uncertainty in the application of results and obstructs innovation. In research, it misguides subsequent studies and restricts collaboration, therefore impacting the advancement and credibility of nursing research and its practical application. For improving the quality of evidence, further thorough experiments are necessary.

This selection bias may be exacerbated by the lack of randomization in many studies, which usually rely on observational data rather than controlled trials^(74)^. In the context of RA, where disease presentation and management can differ significantly among various populations and healthcare settings, this is especially pertinent^(75)^. Because of this discrepancy, it may not be easy to reach firm judgments regarding the relative efficacy of rheumatologist-led versus nurse-led care^(76)^. Furthermore, there may be variations in reported results due to inconsistent application of the instruments used to measure disease activity, such as the Clinical Disease Activity Index (CDAI) or the DAS28^(66)^. The exclusion of particular data sources can substantially alter the results. Studies that do not incorporate qualitative data or patient perspectives might ignore critical aspects of care, including patient satisfaction, treatment compliance, and the overall patient experience. These factors are crucial for a comprehensive understanding of the impact of care models on the lives of patients and can influence the results of treatment^(77)^. Findings may not be as applicable to larger populations if data from various healthcare contexts are excluded, such as public versus private healthcare systems or rural versus urban practices^(78)^. Ethical and regulatory factors also influence the limitations of current research. For example, the necessity of ethical sanction may limit the types of interventions that can be compared in studies and the range of data that can be collected. This is particularly relevant in nurse-led care models, where the scope of practice can vary significantly based on the regulatory framework and region^(79)^.

In healthcare settings, longitudinal data can offer solid proof to back up or disprove these economic assertions, directing resource allocation and policy decisions^(57,58)^. Intense into how nurse-led care influences patient behavior and health outcomes can be gained from longitudinal studies that monitor these variables over time^(80,81)^. Understanding the broader ramifications of incorporating nursing care into rheumatology practices requires knowledge of this information^(82,83)^. The patient experience and the perceived value of nurse-led care over time can be clarified by researchers using rich, qualitative data collected through surveys and interviews conducted at various intervals^(73,80)^.

## CONCLUSION

In conclusion, the current meta-analysis of published RCTs could not identify any substantial differences in the functional status improvements or disease activity of RA patients when comparing RLC and NLC. However, the functional status and disease activity of patients who received NLC demonstrated a more significant improvement than those who received RLC. Per the literature, NLC may offer RA patients more cost-effective treatment and superior clinical outcomes than rheumatologist-led care. Implementing NLC strategies can potentially improve the quality of service, usage of resources, and patient satisfaction of RA patients. NLC may offer RA patients more cost-effective and enhanced clinical outcomes than rheumatologist-led care, as the literature indicates. Applying NLC techniques may enhance RA patients’ overall quality of care, resource efficiency, and patient satisfaction.

## Data Availability

The data are available from the corresponding author on reasonable request.
