# Supplementary material for: Comparing nurse-led and rheumatologist-led care for rheumatoid arthritis patients: a systematic review and meta-analysis of randomized controlled trials
Source: Rev Esc Enferm USP. 2025 Jun 20;59:e20240186. doi: 10.1590/1980-220X-REEUSP-2024-0186en (PMC12186662; doi:10.1590/1980-220X-REEUSP-2024-0186en)
Supplement: Translation

# Translation: Comparación entre los cuidados dirigidos por enfermeras y los cuidados dirigidos por reumatólogos para pacientes con artritis reumatoide: una revisión sistemática y meta-análisis de ensayos controlados aleatórios

**DOI:** 10.1590/1980-220X-REEUSP-2024-0186es

**Authors:** Weiwei Gu, Xiangfu Ding, Xu Han, Junjie Jiang, Hong Li

**Affiliations:** 1 Jilin University, China-Japan Union Hospital, Department of Gastrointestinal Surgery, Changchun, Jilin, China.; 2 Jilin University, The Second Norman Bethune Hospital, Department of Thyroid Surgery, Changchun, Jilin, China.; 3 Jilin University, The Second Norman Bethune Hospital, Department of Rehabilitation, Changchun, Jilin, China.; 4 Jilin University, China-Japan Union Hospital,Traditional Chinese Medicine Department, Changchun, Jilin, China.

**Keywords:** Artritis Reumatoide, Enfermería de Atención Primaria, Reumatólogos, Efectividad, Metaanálisis

## Abstract

**Objetivo:**

Este estudio tiene como objetivo comparar la eficacia de los modelos de atención liderados por enfermeras y por reumatólogos en el manejo de la artritis reumatoide, dado que los estudios previos han mostrado resultados contradictorios.

**Métodos:**

Se realizó una búsqueda en las bases de datos PubMed, Embase y Web of Science para identificar estudios que compararan ambos modelos en pacientes con artritis reumatoide. Los desenlaces analizados incluyeron el Cuestionario de Evaluación de la Salud (Health Assessment Questionnaire, HAQ), el nivel de fatiga, la rigidez, la puntuación de actividad de la enfermedad en 28 articulaciones basada en la proteína C reactiva (DAS28-CRP), la puntuación DAS28, la velocidad de sedimentación globular, la proteína C reactiva y la escala visual analógica de dolor.

**Resultados:**

Siguiendo las directrices de la Colaboración Cochrane, se incluyeron 6 ensayos controlados aleatorizados con un total de 1,093 pacientes. Excepto por la puntuación DAS28, la satisfacción y la duración de la enfermedad, las características basales fueron comparables entre los grupos. El metanálisis mostró una tendencia a la mejora en los parámetros evaluados en el grupo con atención liderada por enfermeras en comparación con el grupo liderado por reumatólogos.

**Conclusión:**

Los resultados del metanálisis indican que los pacientes con artritis reumatoide que reciben atención liderada por enfermeras presentan una tendencia a una mejor evolución en cuanto al estado funcional y la actividad de la enfermedad, en comparación con aquellos atendidos por reumatólogos.

## INTRODUCCIÓN

La artritis reumatoide (AR) es una enfermedad inflamatoria crónica caracterizada por síntomas que incluyen inflamación articular, hiperplasia sinovial y pérdida irreversible de tejido óseo^(1,2)^. Se calcula que en 2020 unos 17,6 millones de personas padecerán artritis reumatoide en todo el mundo^(3,4)^. La artritis reumatoide puede afectar a personas de cualquier edad, incluidos hombres, mujeres y niños; sin embargo, es de dos a tres veces más frecuente en las mujeres. Su incidencia aumenta con la edad, normalmente entre los sesenta y los setenta años^(5)^. La mayoría de los pacientes con AR requieren cuidados médicos y fisioterapéuticos continuos para detener o ralentizar la progresión de esta enfermedad, lo que contribuye a elevar los costes relacionados con el tratamiento^(6)^. Sin embargo, la calidad de vida de millones de personas en todo el mundo se ve afectada negativamente por estos costes, así como por las elevadas tasas de morbilidad y mortalidad relacionadas con la AR^(6)^. Además de los importantes costes indirectos que sufren los pacientes con AR debido a la pérdida de productividad, las nuevas terapias también son muy costosas^(6-8)^. Cuando no reciben un tratamiento eficaz, los pacientes con AR pueden experimentar una disminución del rendimiento funcional, altas tasas de morbilidad y una reducción drástica de la esperanza de vida^(9,10)^. Con el aumento de la prevalencia de las enfermedades crónicas y la prolongación de la esperanza de vida de las personas afectadas, existe una demanda creciente de atención comunitaria y hospitalaria, lo que hace necesario que las enfermeras asuman con frecuencia responsabilidades médicas que tradicionalmente correspondían a médicos o especialistas^(1,2)^. Los cuidados dirigidos por enfermeras (CDE) se consideran un valioso complemento de los cuidados dirigidos por médicos que puede mejorar el bienestar y la calidad de vida de los pacientes que padecen cánceres y enfermedades crónicas^(4)^. Los CDE suponen una estrategia de atención holística centrada en la persona, promovida por la Organización Mundial de la Salud como una faceta clave de los esfuerzos de atención sanitaria de alta calidad^(11,12)^, que contribuye a una mayor satisfacción entre las enfermeras y los pacientes con respecto a la calidad de la atención^(11)^.

El tratamiento hospitalario y clínico de los pacientes con AR es responsabilidad de los reumatólogos. Sin embargo, se prevé que el número de estos especialistas disminuya en el futuro^(1,2)^. En consecuencia, las enfermeras de reumatología se han implicado cada vez más en la realización de tareas que llevan a cabo los reumatólogos, como la prescripción de fármacos y la administración de inyecciones articulares^(1)^. Estas enfermeras también desempeñan funciones clave como miembros de equipos multidisciplinares^(1)^.

Los hallazgos de los 6 ensayos controlados aleatorizados (ECA) no concuerdan con investigaciones previas, lo que sugiere la realización de nuevos análisis. Este estudio evaluó las implicaciones pronósticas de la atención dirigida por enfermeras frente a la dirigida por reumatólogos en pacientes con artritis reumatoide. Un ensayo aleatorizado publicado en 2015 demostró que las mejoras en las puntuaciones del cuestionario de evaluación de la salud (Health Assessment Questionnaire - HAQ) de los pacientes con artritis reumatoide y los costes generales fueron similares, independientemente de si los pacientes recibieron cuidados dirigidos por enfermeras (CDE) o cuidados dirigidos por reumatólogos (CDR)^(1)^. Otros grupos han obtenido resultados contradictorios^(1)^. En total, entre 2020 y 2023 se publicaron 6 ensayos controlados aleatorizados (ECA) centrados en este tema^(13–18)^.

Este artículo tiene como objetivo realizar un análisis de datos y una revisión sistemática de los ensayos clínicos controlados aleatorizados (ECA) existentes para obtener las últimas pruebas médicas basadas en la evidencia para confirmar las ventajas y desventajas de las dos modalidades de tratamiento. El objetivo de este estudio es realizar un análisis exhaustivo y una evaluación sistemática de los datos de los ECA clínicos existentes sobre la AR. Este estudio consolidará los resultados de investigaciones dispares y obtendrá los datos médicos basados en pruebas más recientes y fiables mediante un análisis estadístico meticuloso y una evaluación bibliográfica exhaustiva. Este estudio comparará específicamente la eficacia de las dos modalidades terapéuticas para mejorar las condiciones de los pacientes con AR. Esta evaluación abarcará diversas dimensiones, como el alivio de los síntomas a corto plazo (por ejemplo, la reducción del dolor y la inflamación articulares), el control de la enfermedad a largo plazo (como el mantenimiento de la función articular y el retraso de la progresión radiográfica), la frecuencia y gravedad de las reacciones adversas, y la mejora de la calidad de vida del paciente, para dilucidar sus respectivos puntos fuertes y débiles. Por último, los resultados de este estudio deberían ofrecer una sólida base empírica para la práctica clínica eficaz de la atención al paciente con AR. El desarrollo de planes de tratamiento personalizados ayudará al personal médico a tomar decisiones más científicas y exactas, optimizando los resultados totales del tratamiento y el pronóstico de los pacientes con AR. Esto ayudará a que el campo del tratamiento de la artritis reumatoide mejore y avance y proporcionará directrices de referencia útiles y recomendaciones adicionales para llevar a cabo estudios posteriores relevantes.

Los estudios previos sobre la eficacia de los CDE en comparación con la CDR en pacientes con AR han arrojado resultados inconsistentes. Por lo tanto, este estudio se diseñó para comparar la CDE y la CDR como enfoques para el tratamiento de los pacientes con AR.

## MÉTODO

### Diseño del Estudio

Utilizando como guía la declaración de la Colaboración Cochrane (Preferred Reporting Items for Systematic Reviews and Meta-analysis) 2020, se encontraron estudios que examinaban los costes y la efectividad de CDR y CDE en el tratamiento de los pacientes con AR y se utilizaron para realizar una revisión sistemática y un metanálisis de ECA^(19)^. Este análisis se registró prospectivamente en la base de datos PROSPERO (CRD42023403128), y la lista de comprobación PRISMA 2020 se proporciona en la figura 1.

**Figura 1:** Flujograma del proceso de inclusión y exclusión de la bibliografía.

### Elegibilidad de los Estudios

La elegibilidad para la inclusión en este metanálisis requirió que los estudios cumplieran los siguientes criterios (1) ser ECA; (2) centrarse en pacientes con AR o artritis inflamatoria crónica; (3) comparar el Cuidado Dirigido por Enfermeras CDE y el Cuidado Dirigido por Reumatólogo CDR; (4) informar al menos uno de los siguientes resultados: Puntuación de actividad de la enfermedad para 28 articulaciones (Disease Activity Score for 28 joints -DAS28), DAS28 basada en la proteína C reactiva (DAS28-CRP), Cuestionario de evaluación de la salud (Health Assessment Questionnaire - HAQ), Escala visual analógica (EVA) para el dolor, satisfacción, duración de la enfermedad o resultados clínicos; y (5) proporcionar información adecuada para el cálculo de los valores de la diferencia de medias ponderada (DMP) o el cociente de riesgos (Risk Ratio - RR). En este análisis se excluyeron las investigaciones no publicadas en inglés, los trabajos no publicados, los informes de casos, las cartas, las revisiones, los resúmenes de congresos, los comentarios editoriales y los estudios con pacientes pediátricos. Además, no se incluyeron los análisis epidemiológicos ni los estudios que solo examinaron los costes o la eficacia de CDR o CDE por separado.

P: Pacientes con AR o artritis inflamatoria crónica.

I: CDE

C: CDR

O: DAS 28, DAS 28-CRP, HAQ, EVA Dolor, Satisfacción, Duración de la enfermedad, o Resultados clínicos, etc.

S: ECA

### Estrategia de Búsqueda Bibliográfica

Se realizaron búsquedas en PubMed desde 1964 hasta el 10 de febrero de 2023, en EMBASE desde 1974 hasta el 10 de febrero de 2023 y en Web of Science desde 1945 hasta el 10 de febrero de 2023. Se establecieron como términos de búsqueda las palabras clave relacionadas y el encabezamiento de materia médica: (Nurses’ Practice Patterns) OR (Nurse’s Practice Patterns)) OR (Patrón de práctica de enfermería)) OR (Patrón de práctica de enfermería)) OR (Practice Pattern, Nurse’s)) OR (Practice Patterns, Nurse’s)) OR (Nurse-Led Clinics)) OR (Clinic, Nurse-Led)) OR (Clinics, Nurse-Led)) OR (Nurse Led Clinics)) OR (Nurse-Led Clinic)) OR (nurse-led)) OR («Practice Patterns, Nurses’»[Mesh]) AND (((Rheumatoid Arthritis)) OR («Arthritis, Rheumatoid»[Mesh])) (Archivo suplementario 1). Se examinaron cuidadosamente todas las referencias de investigación pertinentes para identificar estudios adicionales de relevancia potencial. Además, se examinó personalmente la lista de referencias de cada estudio cualificado. Dos investigadores realizaron de forma independiente la búsqueda y evaluación de los estudios incluidos. Todas las diferencias en la revisión bibliográfica se resolvieron mediante consenso.

### Extracción de Datos

La extracción de datos fue realizada de forma independiente por dos investigadores, GWW y HX. Todos los coautores lograron un consenso para abordar cualquier disputa. Los datos obtenidos de los estudios elegidos abarcaron lo siguiente: primer autor, año de publicación, diseño del estudio, orígenes de los participantes reclutados, número de pacientes en cada grupo, edades de los pacientes, proporción de sexos en la población de pacientes y datos clínicos, incluida la duración media de la enfermedad, DAS 28, DAS 28-CRP, HAQ y EVA. Malestar, satisfacción, agotamiento, rigidez, articulaciones inflamadas y sensibles. Para las variables continuas presentadas como medianas y rangos o rangos intercuartílicos, se calcularon en su lugar los valores de media ± desviación estándar con un enfoque matemático previamente validado^(20,21)^. Se intentó contactar con los autores correspondientes de los estudios que carecían de datos completos. Los datos fueron extraídos de forma independiente por dos investigadores, y cualquier disputa fue resuelta por un tercer investigador.

### Análisis Estadístico

Se utilizó Review Manager 5.3 (Cochrane Collaboration, Oxford, Reino Unido) para realizar análisis agrupados. Las variables continuas y categóricas se compararon mediante valores de DMP y RR con sus correspondientes intervalos de confianza (IC) del 95%. Se utilizó la prueba de chi cuadrado (χ^2^) (Q de Cochran) y el índice de inconsistencia (*I* ^2^) para evaluar la heterogeneidad entre los estudios^(22)^, siendo un χ^2^ P < 0,05 o un *I* ^2^ > 50% indicativos de heterogeneidad significativa. También se examinó el impacto de los estudios individuales en los resultados agrupados que mostraban heterogeneidad significativa mediante análisis de sensibilidad unidireccionales, y se inspeccionaron visualmente los gráficos en embudo desarrollados con Review Manager 5.3 para identificar posibles sesgos de publicación. Cuando se identificó heterogeneidad, los valores de DMP o RR agrupados se estimaron mediante un modelo de efectos aleatorios, mientras que en caso contrario se utilizó un modelo de efectos fijos.

## RESULTADOS

### Selección de Estudios

La Figura 1 muestra un diagrama de flujo que describe el procedimiento de selección de los estudios. Primero se obtuvieron un total de 95, 189 y 209 publicaciones potencialmente relevantes de Web of Sciences, EMBASE y PubMed; 218 de ellas se eliminaron por ser duplicados. Tras examinar detenidamente los resúmenes y títulos correspondientes, se seleccionaron 30 de los 277 trabajos para la revisión del texto completo. Finalmente, se seleccionaron 6 estudios con 1.093 pacientes (NLC: 543; RLC: 550) para su inclusión en este metanálisis^(13–18)^, todos ellos ensayos controlados aleatorizados. Para un resumen de las características de estos estudios, véase la Tabla 1 and Figura 1.

**Tabla 1:** Características basales de los estudios incluidos y evaluación metodológica – Changchun, China, 2025.

Autores (año de publicación)	Período de estudio	País (ciudad)	Diseño de estudio	Pacientes (n)	Media de seguimiento (meses)
				Cuidado dirigido por enfermero/Cuidado dirigido por reumatólogo	
Larsson et al. (2014)	2009/10–2011/08	Suecia (Lund)	ECA	47/50	12
Ndosi et al. (2014)	–	Reino Unido (Leeds; Staffordshire; Stoke-on-Trent)	ECA	91/90	–
Larsson et al. (2015)	2009/10–2010/08	Suecia (Lund)	ECA	47/50	12
Hoeper et al. (2021)	2018/01–2018/08	Alemania (Hannover; Osnabruck; Planegg; Erlangen; Hildesheim; Mainz; Bad Kreuznach; Bad Pyrmont)	ECA	111/113	12
Kwok et al. (2022)	-–	China (Hong Kong)	ECA	140/140	24
Wang et al. (2018)	2015/01–2015/11	China (Wenhai)	ECA	107/107	–

ECA, Ensayo controlado aleatorizado

Se utilizó la técnica Cochrane de evaluación de riesgos para evaluar la calidad de la bibliografía incluida. En las 6 investigaciones incluidas se utilizó la asignación por tabla de números aleatorios, lo que se consideró de bajo riesgo. Se utilizó cegamiento para asignar a los individuos a los 6 estudios considerados de bajo riesgo. Las investigaciones restantes se clasificaron como de bajo riesgo y detallaron el proceso preciso de asignación.

Los 6 estudios utilizaron el método del sobre para la ocultación de la asignación, lo que se consideró de bajo riesgo. El doble ciego se mencionó en los seis estudios, y se consideraron de bajo riesgo. Dos estudios^(13,14)^ tuvieron abandonos con las explicaciones correspondientes y se evaluaron como de alto riesgo. Un estudio^(15)^ no había completado los datos de los resultados y se evaluó como de bajo riesgo. Los estudios restantes poseían datos de resultados completos y se evaluaron como de bajo riesgo. Todos los estudios se registraron, lo que indica que se evaluó la notificación selectiva y se determinó que suponía un riesgo bajo. Se determinó que dos estudios^(13,14)^ presentaban otros sesgos y se evaluaron como de bajo riesgo (Figura 2).

**Figura 2:** Gráfico de riesgo de sesgo.

### Características Demográficas Basales

No se observaron diferencias significativas entre estos grupos en cuanto a la edad basal (DMP: –0,18; IC del 95%: –1,26, 0,91; p = 0,75), el sexo (masculino/total, RR: 1,12; IC del 95%: 0,92, 1,37; p = 0,26), la DAS28-CRP (DMP: –0,09; IC del 95%: –0,20, 0,01; p = 0,08), el HAQ (DMP: -0,09; IC del 95%: –0,17, 0,00; p = 0. 05), las puntuaciones de dolor de la EVA (DMP: –0,16; IC del 95%: –0,90, 0,59; p = 0,68), la fatiga (DMP: 0,26; IC del 95%: –0,61, 1,14; p = 0,56), la rigidez (DMP: –1,43; IC del 95%: –7. 17, 4,30; p = 0,62), articulaciones hinchadas (DMP: –0,06; IC 95%: –0,21, 0,09; p = 0,44), o articulaciones sensibles (DMP: –0,09; IC 95%: –0,36, 0,18; p = 0,50). Sin embargo, hubo diferencias significativas en cuanto a los valores basales de la DAS28 (DMP: –0,16; IC del 95%: –0,32, 0,01; p = 0,03), la satisfacción (DMP: 0,28; IC del 95%: 0,10, 0,46; p = 0,002) y la duración de la enfermedad (DMP: 0,60; IC del 95%: 0,02, 1,19; p = 0,04) (Tabla 2).

**Tabla 2:** Características demográficas y clínicas de los estudios incluidos – Changchun, China, 2024.

Resultados	Estudios	Número de pacientes	DMP o RR	95% IC	p-valor	Heterogeneidad			
		/ Cuidados dirigidos por reumatólogo				Chi2	df	p-valor	*I* ^2^ (%)
Edad (años)	6	543/550	–0.18	[–1.26, 0.91]	0.75	3.67	5	0.62	0
Género (masculino)	6	543/550	1.12	[0.92, 1.37]	0.26	1.24	5	0.94	0
DAS 28	4	286/293	–0.16	[–0.32, –0.01]	0.03*	0.25	3	0.97	0
DAS 28-CRP	3	298/303	–0.09	[–0.20, 0.01]	0.08	0.09	2	0.96	0
HAQ	4	325/330	–0.09	[–0.17, 0.00]	0.05	3.20	3	0.36	6
Dolor EVA (0–100mm)	5	496/500	–0.16	[–0.90, 0.59]	0.68	6.81	4	0.15	41
Satisfacción (0–10)	2	158/163	0.28	[0.10, 0.46]	0.002*	0.16	1	0.68	0
Duración de la enfermedad (años)	6	543/550	0.60	[0.02, 1.19]	0.04*	3.65	5	0.60	0
Cansancio (escore EVA)	3	309/310	0.26	[–0.61, 1.14]	0.56	1.83	2	0.40	0
Rigidez	2	198/197	–1.43	[–7.17, 4.30]	0.62	0.40	1	0.52	0
Articulaciones hinchadas	2	218/220	–0.06	[–0.21, 0.09]	0.44	0.01	1	0.91	0
Articulaciones doloridas	2	218/220	–0.09	[–0.36, 0.18]	0.50	0.78	1	0.38	0

* Significancia estadística.

DAS28, puntuación de la actividad de la enfermedad en 28 articulaciones; DMP: Diferencia Media Ponderada; RR, razón de riesgo; IC; Intervalo de confianza; HAQ, Cuestionario de evaluación de la salud; EVA; Escala visual análoga.

### Puntuaciones HAQ

Las puntuaciones HAQ se comunicaron en 3 estudios en los que participaron 558 pacientes (CDE: 278, CDR: 280)^(13,15,17)^. No hubo heterogeneidad significativa (*I* ^2^= 0%, p = 0,90), y no se encontraron diferencias significativas al comparar estos dos grupos de pacientes después del tratamiento (DMP: 0,00; IC del 95%: –0,07 a 0,08; *p* = 0,94) ( Figura 3A).

**Figura 3:** Diagrama de bosque del estudio que evalúa la diferencia en la mejora del HAQ (A), la EVA del dolor (B), la DAS28 (C), la DAS28-CRP (D), la VSG (E), la CRP (F), la fatiga (G) y la rigidez (H) entre el CDR y el CDE en pacientes con AR.

### Puntuaciones de Dolor EVA

En 4 estudios con 716 pacientes (CDE: 356, CDR: 360)^(13,15,16,18)^ se notificaron cambios en las puntuaciones de dolor EVA entre los grupos. No se observaron diferencias significativas en las puntuaciones de la EVA al comparar los grupos de CDE y CDR (RR: –1,36; IC del 95%: –5,17; 2,44; *p =* 0,48), aunque hubo pruebas de heterogeneidad significativa (*I* ^2^ = 93%, *p* < 0,00001) (Figura 3B) y sesgo de publicación (Figura 4B).

**Figure 4:** Diagrama de embudo (Funnel Plot) para el HAQ (A), la EVA Dolor (B), la EVA28 (C), la EVA28-PCR (D), la VSG (E), la PCR (F), la Fatiga (G) y la Rigidez (H) entre el CDR y el CDE en pacientes con AR.

### Puntuaciones DAS28

En 3 ECAs con 601 pacientes (CDE: 298, CDR: 303)^(15–17)^ se comunicaron datos de DAS28-CRP. Los análisis agrupados revelaron mejoras similares en las puntuaciones DAS28-CRP en los grupos de CDE y CDR (DMP: -0,06; IC del 95%: –0,17; 0,04; *p* = 0,25) (Figura 3D), sin pruebas de heterogeneidad (*I* ^2^ = 0%; *p =* 0,56) o sesgo de publicación (Figura 4D).

### Puntuaciones DAS28-CRP

En 3 ECAs con 601 pacientes (CDE: 298, CDR: 303)^(15–17)^ se comunicaron datos sobre la PCR-DAS28. Sin indicios de heterogeneidad (*I* ^2^ = 0%, *p* = 0,56) ni de sesgo de publicación, el análisis agrupado mostró aumentos comparables en las puntuaciones de DAS28-PCR en los grupos de CDE y CDR (DMP: –0,06; IC del 95%: –0,17; 0,04; *p* = 0,25; Figura 3D) (Figura 4D).

### VSG

Los datos relacionados con la velocidad de eritrosedimentación (VSG) se recogieron en 2 estudios en los que participaron 377 pacientes (CDE: 187, CDR: 190)^(15,17)^. Los análisis agrupados no revelaron diferencias significativas en los valores de VSG en el grupo de CDE (DMP: –1,17; IC del 95%: –3,55; 1,21; *p* = 0,34), y no hubo pruebas de heterogeneidad significativa (*I* ^2^ = 0%; *p* = 0,88) (Figura 3E). Se detectó un ligero sesgo de publicación mediante la inspección visual de los gráficos en embudo (Figura 4E).

### PCR

Los niveles de proteína C reactiva (PCR) se analizaron en 2 estudios en los que participaron 377 pacientes (CDE: 187; CDR: 190)^(15,17)^. Los análisis agrupados no revelaron un aumento significativo de los niveles de PCR en el grupo de CDE (RR: –0,40; IC del 95%: –1,52; 0,72; *p* = 0,49).Se detectó una heterogeneidad significativa (*I* ^2^ = 83%, *p* = 0,01) (Figura 3F) y un ligero sesgo de publicación (Figura 4F).

### Fatiga

La fatiga fue un criterio de valoración analizado en 3 estudios en los que participaron 619 pacientes (CDE: 309; CDR: 310)^(13,16,18)^. Se detectó una heterogeneidad significativa (*I* ^2^ = 98%, *p <* 0,00001) (Figura 3G) en los análisis agrupados, que revelaron una tendencia hacia una mejora de la fatiga en el grupo de CDE (RR: –5,20; IC del 95%: –13,74; 3,35; *p* = 0,23). Los gráficos en embudo revelaron sesgo de publicación y heterogeneidad detectada (Figura 4G).

### Rigidez

La rigidez se analizó en 2 estudios en los que participaron 395 pacientes (CDE: 198; CDR: 197)^(13,18)^. Los análisis agrupados no revelaron mejoras significativas de la rigidez en el grupo de CDE en relación con el grupo de CDR (RR: –9,64; IC del 95%: –19,40; 0,12; *p =* 0,05), habiéndose detectado una ligera heterogeneidad (*I* ^2^ = 39%; *p* = 0,20) (Figura 3H).

### Análisis de Sensibilidad

Se emplearon análisis de sensibilidad unidireccionales para analizar las comparaciones de los resultados agrupados DAS28, puntuación de dolor EVA y cansancio mediante la exclusión sistemática de estudios individuales del análisis agrupado y la evaluación del efecto sobre el riesgo relativo (RR) agrupado. Los valores de RR permanecieron constantes tras excluir cualquier estudio específico para los tres resultados (Figura 5A–C). Sin embargo, cuando se excluyó el estudio realizado por Wang et al. en 2018^(18)^ , ya no se detectó heterogeneidad significativa para los análisis agrupados de fatiga (*I* ^2^ = 0%, p = 0,56), DAS28 (*I* ^2^ = 0%, p = 0,91) o puntuaciones de dolor EVA (*I* ^2^ = 0%, p = 0,54), lo que indica que este estudio fue la principal fuente de heterogeneidad.

**Figura 5:** Análisis de sensibilidad de DAS28 (A), Fatiga (B) y Dolor EVA (C) entre CDR y CDE para pacientes con AR.

## DISCUSIÓN

El presente metaanálisis se desarrolló para comparar la eficacia de los CDR y los CDE para los pacientes con AR en función de las diferencias en los resultados, incluidos los valores de HAQ, DAS28, DAS28-CRP, VSG, PCR, fatiga, rigidez y puntuación EVA del dolor entre estos grupos^(23-27)^. El tratamiento de los pacientes con AR basado en los CDE se ha relacionado anteriormente con una mejora de las opiniones sobre su atención y de los resultados clínicos^(28–31)^. No se dispone de datos suficientes para llegar a conclusiones definitivas sobre la viabilidad, la eficacia o la relevancia de la NLC para esta población de pacientes^(28)^. El RLC y el NLC parecen ser enfoques comparables y rentables para el tratamiento de los pacientes con AR. Por lo tanto, el presente estudio fue el análisis conjunto más reciente y de mayor envergadura centrado en este tema, que sintetizó los datos de 6 ECA en los que participaron 1.093 pacientes de ECA y produjo varios datos importantes. Estos análisis revelaron que los pacientes que se sometieron a RLC mostraron una tendencia hacia mejores resultados clínicos que los pacientes que se sometieron a NLC. Sin embargo, no hubo diferencias significativas en los resultados entre estos dos grupos. La AR es una enfermedad inflamatoria crónica que contribuye a elevar las tasas de mortalidad entre los pacientes afectados^(32)^, además de causar efectos adversos significativos en el estado funcional y la calidad de vida debido a síntomas que incluyen hinchazón generalizada, sensibilidad y un mayor riesgo de fracturas^(33,34)^. Se estima que la esperanza de vida de los pacientes con AR se reduce entre 3 y 10 años con respecto a la población general, con un riesgo ~50% mayor de muerte prematura^(35)^. Aunque aún se desconoce el fundamento etiológico exacto de la AR, los factores genéticos y ambientales contribuyen a su incidencia^(36,37)^. Se han relacionado muchos factores diferentes con la aparición y la progresión de la AR, como la edad, el sexo, los factores genéticos, los niveles hormonales, el origen étnico, los factores socioeconómicos, las infecciones, el hábito tabáquico, la urbanización y la exposición a contaminantes^(32,38)^. El HAQ constituye un medio eficaz para evaluar la calidad de vida y el estado funcional de los pacientes con AR^(37,39)^, por lo que esta herramienta analítica de referencia se ha traducido a numerosos idiomas^(40,41)^. El HAQ de 20 ítems se diseñó como un instrumento amplio para evaluar poblaciones de pacientes en lugar de dirigirse únicamente a pacientes con AR. Contiene ítems sobre 8 dominios utilizados para evaluar las actividades de la vida diaria, como caminar, alcanzar objetos, vestirse, agarrar objetos y comer^(41,42)^. Esta escala se ha establecido como una herramienta fiable y válida para la evaluación de pacientes con AR^(43)^. En consecuencia, se utilizó para evaluar el estado funcional de los pacientes con AR, demostrando mejoras comparables en el estado funcional de los que tenían CDR o CDE.

La DAS proporciona una herramienta cuantitativa que puede utilizarse para monitorear la actividad de la enfermedad y las respuestas terapéuticas en pacientes con AR^(44)^. Esta escala incorpora datos sobre la sensibilidad articular, la inflamación articular, los valores de VSG y los niveles de PCR, junto con una visión global del estado de salud general del paciente^(45)^. La DAS se utiliza a menudo en la clínica para evaluar la gravedad de la AR, aunque no se ha establecido como la herramienta de referencia para su uso en este contexto^(46)^. Una variable no puede englobar por completo la actividad de la enfermedad en un determinado paciente con AR^(47)^. No obstante, en los estudios clínicos y en la práctica clínica habitual se ha utilizado el DAS/DAS28 para evaluar la actividad de la enfermedad de los pacientes con AR tanto individualmente como en grupos. En el presente estudio se utilizaron los parámetros DAS28 y DAS28-CRP para evaluar la eficacia de las técnicas de intervención basadas en RLC y NLC, y los resultados mostraron que ambos mejoraron de forma comparable en los dos grupos de pacientes con AR. Anteriormente no se habían descrito diferencias significativas en las puntuaciones DAS28 de los pacientes con AR sometidos a CDE en relación con las asociadas a CDR a los 12 meses^(15,48,49)^. Sin embargo, se ha informado de que el CDE muestra una superioridad significativa a los 2 años^(48)^.

En algunos estudios se ha observado que las puntuaciones DAS28 mejoran significativamente en los pacientes con artritis inflamatoria a los 9 meses o en los pacientes con AR a los 12 meses tratados con CDE en comparación con CDR^(18,50)^. En cambio, otros estudios no han detectado diferencias significativas a los 13 meses^(18)^, 21 meses^(50)^ o 24 meses^(51)^. Se observaron valores similares de VSG, PCR y puntuación EVA del dolor en ambos grupos. Al calcular las puntuaciones DAS o DAS28, pueden utilizarse los niveles de PCR en lugar de los valores de VSG^(52)^. La PCR proporciona una mayor precisión como indicador de la actividad inflamatoria y aumenta más rápidamente tras la exposición a estímulos inflamatorios^(53)^. Chew et al.^(54)^ observaron que la actividad de la enfermedad en la mayoría de los pacientes con AR se mantenía estable entre los pacientes que recibían CDE, con ligeros aumentos en las puntuaciones medias de la DAS28. Se observaron mejoras comparables en la rigidez y la fatiga en ambos grupos, lo que contrasta con los resultados de un metaanálisis anterior, que indicaba que las CNF no podían aliviar la fatiga en los pacientes con AR^(31)^. Sin embargo, no se observaron diferencias significativas en los resultados al comparar los grupos de CDE y CDR^(55)^. La base de estos resultados sigue siendo incierta. No obstante, la comunidad médica reconoce cada vez más la importancia de los CDE, que presentan ventajas potenciales para mitigar el cansancio de los pacientes con AR mediante tratamientos educativos y de apoyo psicosocial dirigidos por enfermeras. Los CDE aportan ventajas como una mayor continuidad de los cuidados y la posibilidad de que los pacientes accedan a ellos permaneciendo cerca de su domicilio^(56)^. En personas con enfermedades crónicas, se han establecido modelos de atención basados en los CDE en EE.UU., Canadá y Australia^(13)^. Las estrategias de NLC permiten a los reumatólogos trabajar de forma más eficaz al tiempo que experimentan niveles de estrés más bajos^(15)^. Un ECA multicéntrico informó de que la NLC era superior a la RLC en cuanto a satisfacción del paciente con AR y costes de consulta^(13)^. Además, en comparación con los pacientes que recibieron RLC, los datos actuales indican que los pacientes que recibieron NLC mostraron mayores ganancias en su estado funcional y resultados clínicos. En el caso del estudio de Wang et al., varios factores pueden contribuir a la variabilidad observada. Muchos factores pueden influir en la heterogeneidad de los resultados, sobre todo en medidas como el dolor EVA, la fatiga y la DAS28.

La investigación realizada por Wang et al. estableció unos criterios de inclusión y exclusión bien definidos. Se excluyó a los pacientes que habían sido diagnosticados recientemente, mostraban una discapacidad grave o presentaban una enfermedad inestable. Este proceso de selección puede haber dado lugar a una población de pacientes específica con características únicas, que podrían diferir de las de otros estudios, lo que podría influir en los resultados y contribuir a la heterogeneidad.

La aplicación de herramientas como la DAS28 y la EVA para evaluar el dolor y la fatiga puede mostrar variabilidad en la interpretación y el uso. A pesar de la formación, los evaluadores pueden variar sus métodos para contar las articulaciones afectadas en la DAS28 o evaluar el dolor y la fatiga en la EVA. Esto puede dar lugar a variaciones en las puntuaciones registradas y contribuir a la heterogeneidad en relación con otros estudios. La estandarización de las terminologías en el tratamiento de la artritis reumatoide puede mejorar la comunicación, la comparabilidad de las investigaciones y los resultados de los pacientes para los profesionales sanitarios. La estandarización es esencial debido a la evolución de las funciones del personal de enfermería en la atención reumatológica, que requiere definiciones y normas claras para los modelos de CDE y CDR^(16,18,57,58)^.

El estudio se llevó a cabo dentro de un sistema sanitario en China, que podría mostrar características distintas, incluyendo variaciones en la disponibilidad y accesibilidad de la medicación, diferencias en la organización de los servicios sanitarios y factores culturales que afectan a las interacciones paciente-proveedor. Los factores contextuales pueden interactuar con los modelos de CDE y CDR de forma diferente a otras regiones o sistemas sanitarios, dando lugar a heterogeneidad en los hallazgos en relación con estudios realizados en contextos diferentes.

En resumen, la variabilidad en los hallazgos del estudio de Wang et al., en particular en lo que respecta a medidas como el dolor EVA, la fatiga y la DAS28, puede ser el resultado de una compleja interacción de diversos factores asociados con el diseño del estudio, la implementación de la intervención, la recopilación de datos y el contexto sanitario más amplio. La comprensión de estas posibles fuentes de heterogeneidad mejora la interpretación de los resultados y facilita las comparaciones con otros estudios de campo.

A pesar de la agregación de datos de múltiples estudios, la ausencia de significación estadística en resultados como la fatiga y la rigidez en el metanálisis puede deberse a diversos factores clínicos y estadísticos. La fatiga y la rigidez en la AR son síntomas multifactoriales. Pueden estar influidos no sólo por la actividad de la enfermedad, sino también por otros factores, como las comorbilidades, el estilo de vida y los factores psicológicos. Los diferentes estudios podrían haber tenido proporciones variables de pacientes con estos factores de confusión, lo que podría haber enmascarado el verdadero efecto de los modelos NLC o RLC sobre la fatiga y la rigidez.

Las técnicas empleadas para cuantificar la rigidez y la fatiga en los distintos ensayos pueden haber introducido una heterogeneidad sustancial. Las medidas de la duración de la rigidez y la EVA para el cansancio son subjetivas y vulnerables a los sesgos en los informes y la percepción de los pacientes. Es posible que en otros estudios se utilizaran versiones ligeramente diferentes de estos instrumentos de evaluación o instrucciones para los pacientes sobre cómo valorar los síntomas. Esta imprecisión en la medición puede haber disminuido la posibilidad de encontrar una diferencia estadísticamente significativa y aumentado la variabilidad de los datos. Wang et al.^(59)^ realizaron ensayos aleatorizados en pacientes chinos con AR para evaluar la eficacia clínica y la rentabilidad de la NLC en comparación con la RLC. Sus resultados preliminares indicaron que los pacientes con AR tratados mediante CDE pueden tener mejores resultados clínicos y una atención más rentable. Un estudio^(60)^ implantó un sistema de revisión iniciado por el paciente para personas con AR, que mejoró la satisfacción del paciente y el uso de recursos. En un estudio^(61)^ se compararon los resultados y la relación coste-eficacia de los CDE comunitarios con la atención ambulatoria dirigida por reumatólogos. El estudio presentó los resultados de los análisis de regresión y sugirió que el CDE en la comunidad podría ser una opción coste-efectiva para el tratamiento de los pacientes con AR.

Además, Lopatina et al.^(62)^ compararon la utilización de recursos sanitarios y los costes en pacientes estables con AR bajo modelos de prestación de cuidados dirigidos por enfermeras y por reumatólogos. La bibliografía indica que los CDE pueden proporcionar resultados clínicos superiores y una atención coste-efectiva a los pacientes con AR en comparación con la atención dirigida por reumatólogos. La adopción de estrategias de NLC podría mejorar la satisfacción del paciente, la utilización de recursos y la calidad general de la atención para los pacientes con AR. Se aconseja seguir investigando y aplicando estrategias, especialmente en África y Oriente Medio^(29)^.

Este estudio presenta varios puntos fuertes y limitaciones. Es importante destacar que este análisis se basó en 6 ECA en los que los pacientes fueron asignados aleatoriamente a grupos asistenciales y se disponía de datos asistenciales completos^(13-18)^, lo que refuerza la credibilidad de las conclusiones resultantes. Sin embargo, los intervalos de seguimiento de estos estudios diferían sustancialmente, lo que podría dar lugar a cierto grado de sesgo a la hora de interpretar los resultados agrupados. Este estudio utilizó bases de datos como PubMed, Embase y Web of Science para buscar únicamente artículos publicados. Este estudio no puede asegurar la recolección de artículos no publicados, grises y presentados actualmente para un análisis exhaustivo. Por lo tanto, las imprecisiones de los datos pueden requerir un examen y rectificación adicionales en futuras investigaciones. Las investigaciones futuras deberán incluir experimentos adicionales de alta calidad que tengan en cuenta los intervalos de seguimiento, en función de la publicación de más estudios sobre este tema. Este metanálisis no incluyó estudios no publicados, lo que puede haber llevado a la omisión de datos relevantes.

El conocimiento de los matices culturales permite al personal de enfermería adaptar las intervenciones de forma eficaz, lo que aumenta la implicación del paciente y su adherencia al tratamiento, mejorando así los resultados sanitarios^(63)^. Los modelos de cuidados dirigidos por enfermeras pueden integrarse más fácilmente en el tratamiento de la AR en países con sistemas sanitarios sólidos, facilitando estrategias de gestión integral que abarquen el apoyo, el monitoreo y la educación. Al examinar estas variaciones, los investigadores pueden identificar las mejores prácticas que pueden adaptarse para su aplicación en diversos entornos sanitarios, garantizando que los cuidados dirigidos por enfermeras se personalicen para satisfacer las necesidades específicas de la población^(64)^. Los pacientes más jóvenes podrían preferir métodos tecnológicamente más avanzados, como las consultas de telesalud con enfermeras. Sin embargo, los pacientes de más edad podrían beneficiarse más de las reuniones cara a cara con los reumatólogos. Conocer estas preferencias puede ayudar a crear planes de atención individualizados que mejoren los resultados y la satisfacción de los pacientes^(65,66)^. Al analizar estos factores, los investigadores pueden comprender mejor cómo interactúa el nivel socioeconómico con los modelos asistenciales para influir en los resultados de los pacientes^(67)^. Comprender las variaciones regionales facilita la asignación de recursos y garantiza que los sistemas sanitarios estén equipados para satisfacer las necesidades específicas de sus poblaciones^(68)^.

La disponibilidad de recursos en los distintos sistemas sanitarios difiere significativamente. En un sistema sanitario rico en recursos, el modelo de CDE tiene el potencial de integrar eficazmente equipos multidisciplinares, incluidos fisioterapeutas y dietistas, mejorando así la atención integral al paciente e influyendo positivamente en su pronóstico. En un sistema sanitario con recursos limitados, el modelo de CDR puede reforzar las decisiones de diagnóstico y tratamiento debido a la autoridad profesional de los médicos; sin embargo, puede quedarse corto a la hora de ofrecer intervenciones no farmacológicas adecuadas, como la educación del paciente y el apoyo psicológico.

Curiosamente, en un metaanálisis no se observaron diferencias apreciables entre los resultados del seguimiento dirigido por médicos y los del seguimiento dirigido por enfermeras, lo que indica que los cuidados dirigidos por enfermeras pueden tratar con éxito a los pacientes con AR con baja actividad de la enfermedad, al igual que los cuidados tradicionales dirigidos por reumatólogos^(69,70)^. Un estudio pragmático, no aleatorizado, evaluó la relación coste-eficacia de los CDE basados en la comunidad frente a los CDR. Los resultados indicaron que el CDE se asoció a unos costes anuales más bajos, manteniendo al mismo tiempo los resultados clínicos. En concreto, el coste anual total de la atención reumatológica para el grupo de CDE fue significativamente inferior al del grupo de CDR, lo que sugiere que los CDE proporciona un tratamiento eficaz con una reducción de los gastos médicos^(61,71)^. También se ha observado que el uso de medicamentos antirreumáticos modificadores de la enfermedad (Disease-modifying antirheumatic drugs - DMARD) está creciendo; entre 1998 y 2009, su uso aumentó significativamente. Este patrón indica un mayor movimiento hacia enfoques terapéuticos agresivos que buscan una baja actividad o remisión de la enfermedad, esenciales para los resultados a largo plazo de los pacientes^(72,73)^. Existen algunas limitaciones a la hora de excluir las bases de datos de enfermería como referencia. Pueden perderse hallazgos de investigaciones en profundidad con puntos de vista distintos o preocupaciones enfermeras particulares, y es posible que otras bases de datos extensas no incorporen con frecuencia estos hallazgos. Las bases de datos de enfermería ofrecen presentaciones sucintas de casos específicos, experiencias de enfermería y otra información pertinente para la práctica enfermera. La ausencia de estos elementos puede conducir a una comprensión incompleta de ciertos fenómenos enfermeros y dificultar la capacidad de abordar eficazmente la diversidad y especificidad dentro del campo de la enfermería, comprometiendo la exhaustividad y aplicabilidad de los metaanálisis relativos a temas de investigación en enfermería. El sesgo de publicación puede estar indicado por un gráfico en embudo que muestre una clara asimetría, evidenciada por la ausencia de puntos de estudio específicos en un lado. Existen varias estrategias para mitigar el impacto del sesgo de publicación. Esto implica identificar cuidadosamente los estudios no publicados para subsanar cualquier deficiencia, por ejemplo explorando el registro de ensayos clínicos o poniéndose en contacto con los autores del estudio para obtener datos no publicados. Una vez excluidas las investigaciones potencialmente sesgadas, realizar un análisis de sensibilidad para evaluar la solidez de los resultados. La eficacia y las limitaciones de los seis ensayos controlados aleatorizados que evalúan los efectos de CDE y CDR sobre el pronóstico de los pacientes con AR pueden analizarse de forma más exhaustiva mediante el examen detallado de la diversidad de la investigación y el sesgo de publicación mencionados anteriormente, proporcionando referencias valiosas para la práctica clínica y las investigaciones futuras. No publicar el protocolo de la RSL conduce a la falta de transparencia y a una reproducibilidad reducida. Aumenta los riesgos de sesgo, como la notificación selectiva. En la práctica clínica, genera incertidumbre en la aplicación de los resultados y obstaculiza la innovación. En la investigación, orienta mal los estudios posteriores y restringe la colaboración, por lo que repercute en el avance y la credibilidad de la investigación en enfermería y en su aplicación práctica. Para mejorar la calidad de las pruebas, son necesarios más experimentos exhaustivos.

Este sesgo de selección puede verse exacerbado por la falta de aleatorización en muchos estudios, que suelen basarse en datos observacionales y no en ensayos controlados^(74)^. En el contexto de la AR, en el que la presentación y el tratamiento de la enfermedad pueden diferir significativamente entre distintas poblaciones y entornos sanitarios, esto es especialmente pertinente^(75)^. Debido a esta discrepancia, puede que no sea fácil emitir juicios firmes sobre la eficacia relativa de los cuidados dirigidos por reumatólogos frente a los dirigidos por enfermeras^(76)^. Además, puede haber variaciones en los resultados comunicados debido a la aplicación incoherente de los instrumentos utilizados para medir la actividad de la enfermedad, como el índice clínico de actividad de la enfermedad (CDAI) o la DAS28^(66)^. La exclusión de determinadas fuentes de datos puede alterar sustancialmente los resultados. Los estudios que no incorporan datos cualitativos o perspectivas de los pacientes pueden ignorar aspectos críticos de la asistencia, como la satisfacción del paciente, el cumplimiento del tratamiento y la experiencia general del paciente. Estos factores son cruciales para una comprensión global del impacto de los modelos de atención en la vida de los pacientes y pueden influir en los resultados del tratamiento^(77)^. Los resultados pueden no ser tan aplicables a poblaciones más amplias si se excluyen los datos procedentes de diversos contextos sanitarios, como los sistemas sanitarios públicos frente a los privados o las prácticas rurales frente a las urbanas^(78)^. Los factores éticos y normativos también influyen en las limitaciones de la investigación actual. Por ejemplo, la necesidad de una sanción ética puede limitar los tipos de intervenciones que pueden compararse en los estudios y la variedad de datos que pueden recolectarse. Esto es especialmente relevante en los modelos de cuidados dirigidos por enfermeras, en los que el alcance de la práctica puede variar significativamente en función del marco normativo y de la región^(79)^.

En los entornos sanitarios, los datos longitudinales pueden ofrecer pruebas sólidas para respaldar o refutar estas afirmaciones económicas, orientando la asignación de recursos y las decisiones políticas^(57,58)^. Los estudios longitudinales que monitorean estas variables a lo largo del tiempo pueden ayudar a comprender mejor cómo influyen los cuidados dirigidos por enfermeras en el comportamiento de los pacientes y en los resultados sanitarios^(80,81)^. Para comprender las ramificaciones más amplias de la incorporación de los cuidados de enfermería a las consultas de reumatología es necesario conocer esta información^(82,83)^. La experiencia del paciente y el valor percibido de los cuidados dirigidos por enfermeras a lo largo del tiempo pueden ser aclarados por los investigadores utilizando datos cualitativos y ricos, recolectados mediante encuestas y entrevistas realizadas a diversos intervalos^(73,80)^.

## CONCLUSIÓN

En conclusión, el presente metanálisis de los ECA publicados no pudo identificar diferencias sustanciales en las mejoras del estado funcional o la actividad de la enfermedad de los pacientes con AR al comparar los CDR y los CDE. Sin embargo, el estado funcional y la actividad de la enfermedad de los pacientes que recibieron CDE demostraron una mejoría más significativa que los que recibieron CDR. Según la bibliografía, los CDE pueden ofrecer a los pacientes con AR un tratamiento más rentable y resultados clínicos superiores que la atención dirigida por reumatólogos. La implementación de estrategias de CDE puede mejorar potencialmente la calidad del servicio, el uso de recursos y la satisfacción de los pacientes con AR. Los CDE pueden ofrecer a los pacientes con AR un tratamiento más rentable y unos resultados clínicos superiores que la atención dirigida por reumatólogos, tal y como indica la bibliografía. La aplicación de técnicas de CDE puede mejorar la calidad general de la atención, la eficiencia de los recursos y la satisfacción de los pacientes con AR.

## Data Availability

Los datos están disponibles del autor correspondiente en petición razonable.
